# Patriotism and the Nurse-Training Schools

**Published:** 1915-10-23

**Authors:** 


### PATRIOTISM AND THE NURSE-TRAINING SCHOOLS.

### I. EXAMPLES IN GREAT BRITAIN AND IRELAND.

The Training Schools for Nurses throughout the kingdom have shouldered their burden in the great war with a quiet enthusiasm very little understood in the nation at large. The deep spirit of dedication to the service of humanity, which wells up (we may thank God for it) in all our institutions for the sick, has flowed out inexhaustibly in the calamity which has fallen upon the world. That the universal outpouring of zeal in the cause of suffering humanity was untainted by lust for novelty and excitement has been already abundantly proved among members of the nursing profession. Not merely has it been revealed in the patient continuance at their posts of the women who were needed at home, but it has been continuously demonstrated during the past fifteen months in every theatre of war, where hard tasks of a monotonous and burdensome character have been encountered with a gallantry which defies description. In recording the sacrifices of nurses in the interests of their country it is hard to say which are most to be admired, those who went or those who stayed. Perhaps the situation is most happily summed up in the words of a matron who records the fact that many of her nurses were doing good work among the troops, but that none had yet been decorated for distinguished service: "Each one is a heroine to me."

We have thought it well that a special effort should be made to gather together the records of this inspiring time, to probe such sources of information as are open to us and to preserve the names of our sisters fighting the good fight no less bravely than British soldiers wherever the struggle rages for the liberty of mankind. The patriotic services rendered by the nurse-training schools of the country fall under four headings, and it is sufficient to observe that had these institutions failed the country in any one of these particulars grave disaster must have fallen upon our troops. The first and greatest service they rendered was the efficient preparation of nurses to meet this crisis, a preparation which has been in progress during many years past. Next, there has been the willing surrender of many valuable members of the staff for war service, necessitating a swift adaptation of the machine to meet altered circumstances. Thirdly, there has been the surrender of some part of the institution for the reception of the wounded. And lastly must be taken into account the amazing resourcefulness with which emergency training has been improvised for Voluntary Aid Detachments and other women needed to reinforce the supply of regular probationers. Taken together these four achievements sufficiently attest the very high pitch of efficiency reached by the nurse-training schools at this stage in their development. The fact that they have been able to meet so heavy a strain in various directions witnesses to their force as living entities capable of expansion and growth, and we cannot forbear the hint that this constitutes in itself a startling testimony to the benefits of freedom from a rigid code of uniform regulations.

In presenting examples of patriotism in the nurse-training schools we propose to confine ourselves to no one section of these institutions, nor to any one part of the United Kingdom. The world-renowned hospitals have hardly done better in their sphere and in proportion than some obscure institutions in places seldom visited by the general public.

### ST. GEORGE'S HOSPITAL.

From the first St. George's lent itself to the task of fitting women to be useful in the coming emergencies plainly to be foreseen in the opening weeks of war. The unstinted devotion with which this hospital met the crisis is revealed in the proportion of the staff which has been spared for war duty. The nurses whose names we append number thirty-two, and they form 23 per cent. of the total staff previous to the outbreak of war. When it is remembered that in normal times few hospitals have more than 25 per cent. of fully-trained nurses on their staff the extent of this surrender can be to some extent appreciated. The members of the staff now on duty under the Army and Red Cross are the following: Nurses Morrison, Johnstone, Hatton, Maltby, Whittuck, Jeffrey, Walsh, Rice-Oxley, Hunter, Knights, Brownrigg, B. Robinson, Moss, Young, Newton, Seymour, Rowe, Barry, Tyrie, Pike, Meggitt, Sturt, Thomas, Cape, Carter, Bish, Shard, Hearn, Egles, Nairne, Stoddart, Jones. Nurse Mary White obtained the medal of the "Legion of Honour," while working for the Belgian Red Cross, for rescuing wounded from the trenches under fire.

### THE METROPOLITAN HOSPITAL.

A very close bond unites the members of this training school, and they keep their matron well informed of their movements after they leave. In consequence we are able to give the names of forty-two Metropolitan sisters and nurses tending the wounded in different parts of the world. About six of these were not actually trained at the hospital, but were closely identified with it as ward-sisters. Eleven out of the number, or 20 per cent. of the staff, went direct from the institution. *Q.A.I.M.N.S.*: Sisters Davis, Hodgins, Medforth, McCabe, McClelland, Harrison. *Q.A.I.M.N.S.R.*: Sisters Allen, M. Barwell, E. Barwell, Branford, Carey, Chapman, Deacon, Hanhart, Hebbes, Lawrence, Pike, Stephenson, Sykes, Lubt, Thomas, Watts, Woodhouse, Walker. *Q.A.R.N.N.S.* (and Reserve): Sisters Bayly, Hawes, Chamberlain. *Red Cross, France*: Sisters Kemmey, Maxwell-Stuart,

Ricketts, Taylor. *Serbia*: Sisters Blackstock, Chaplin, Piper, Stokes, Simpson. *Egypt*: Sisters Connell, Burton, Tate. *Malta*: Sisters Cheverton, Lonsdale. *Africa*: Sister Stuart. In addition, there are twenty-seven sisters and nurses in military and Territorial hospitals at home. The list gives a stirring impression of the extent to which the members of a comparatively small training school are making their mark in different directions.

### ROYAL UNITED HOSPITAL, BATH.

Out of a total nursing staff of thirty-eight no fewer than thirteen, or 34 per cent., have been surrendered for duties in connection with the war. The following are those now serving:—*Q.A.I.M.N.S.R.*: Sister Dawson (night superintendent), in France since August 1914; Miss E. Parkinson, now in Egypt; Sister A. W. Willis (assistant matron), at Queen Mary's Military Hospital, Whalley. *T.F.N.S.*: Nurses Joyce, Cox, Alves, Brewin, Fisher, O'Sullivan, Olds. *Q.A.I.M.N.S.*: Sister Parkinson, joined just before the war. Two other members of the Q.A.I.M.N.S.—viz. Sister Hilda N. Drage and Sister Cordelia Mackay—received their training at the Bath Hospital and have recently reflected glory on their *Alma Mater* by the fact that they have been mentioned in dispatches for fine services rendered in France since the beginning of the war.

### ROYAL INFIRMARY, LIVERPOOL.

A good contingent, amounting to close on 25 per cent. of the nursing staff, have joined the Navy and Army for hospital and other nursing duties in connection with the war. They may be classified as follows:—*Admiralty*: At Malta, Sister Swift, Nurse Ainsworth. *R.N. Hospital, Plymouth*: Nurses Hutton, Macphie, Hunt. *Haslar*: Nurses Hercus and Pickard. *Military—Expeditionary Force in France*: Sisters Stanley, Sinclair, Hardy, Taylor, Adams, Macfarlane; Nurses Garner, Miller, Wight, Johnson, Matthews. *Egypt*: Sister Gregson. *Hospital Ships*: Nurses Royal and Close. *Aldershot*: Nurse A. Turner.

### BOOTLE BOROUGH HOSPITAL.

This is a small hospital with a staff numbering only twenty-four. Yet eight out of this small number are now on war service, and have left direct from the institution this year for that purpose. First on the list should be mentioned Sister Stone, matron of the Church Army Hospital of St. Jean, Caen. In recognition of the excellent work she has accomplished here she has received the medal of "La Société Française de Secours aux Services Militaires." Other members of the staff are:—*Q.A.I.M.N.S.*: Sister Arnold, who has sailed for the Dardanelles; Sister Urquhart; Nurse Cunningham. Nurse Lysaght is on the Transport Service, Nurses Hocknell and Wild are on the Navy List, and Nurse Parry is on the Territorial Force. This is indeed a good record.

### ROYAL INFIRMARY, CHESTER.

Seven members of the nursing staff—15 per cent. of the total number—are at present actively engaged with the Army Nursing Service. Their names are as follows: Sisters Warburton, Boulton, Pickard, Hargraves; Nurses Richardson, Leman, Sparkes. It should be mentioned that in addition to the twenty beds which have been used for sick and wounded soldiers since the beginning of the war, the Chester Infirmary will shortly have accommodation for a further fifty beds; whilst in two months or so the military accommodation will be increased by a still further fifty beds, making in all 120 military beds, with ninety civilian beds. Under these circumstances the staff may almost be reckoned *in toto* as engaged in war work.

### COUNTY AND CITY ROYAL INFIRMARY, PERTH.

Six members of the nursing staff out of a total of twenty-five—24 per cent.—have been called up for military duty since the beginning of the war. Sister Fife has gone out to Cairo. Nurses Lunan and Gordon are with the Expeditionary Force, somewhere in France. Sisters Wilson and Leslie, and Nurse Henderson, are with the Territorial Nursing Service. Perth is one of the hospitals which have nobly relinquished their matrons for the good cause. Miss Bowhill, matron and superintendent of nurses, is at present in charge of the Scottish Women's Hospital at Kragujevatz, Serbia, where she has been over six months, doing splendid work of which the world has heard rumours but little more. Miss E. T. Ferguson, who was mentioned in dispatches, and to whom was awarded the Royal Red Cross, was trained at Perth.

### DUMFRIES AND GALLOWAY ROYAL INFIRMARY.

Among the nurses who have gained distinction since the war began, Miss M. Stimson, mentioned in Sir John French's dispatches, was trained at the above hospital. Sister E. Stimson is at present in charge of a hospital train in France. Nurses Simson and Portious have been called up to Netley. *Q.A.I.M.N.S.R.*: Nurses Harkness, Nicholson, Kean, Armstrong, Mitchell, McGan. *Territorial Hospital*: Nurses Gourlay and Bell. In *Red Cross Hospitals*: Nurses Brechin, Smith, Dodson, Haston, Hughes, Gordon, McGregor. Sister Barron, now in the East, was night sister at Dumfries when called up for duty, but was trained at Aberdeen. Sister Mair, who was trained at the Royal Infirmary, Edinburgh, also went from Dumfries to take up work in France; and the hospital sustained the loss of a third sister in Sister Gilmour, who was trained at Aberdeen, and left at the outbreak of war to take up work at the 1st Territorial Hospital in that city.

### ROYAL INFIRMARY, CORK.

Eight of the staff of this infirmary, about 19 per cent. of the total, are now employed in connection with the war. Nurse Sloyd is in Alexandria; Nurse May O'Callaghan is in Malta; Nurse E. O'Sullivan is in a field hospital in France; and Nurses Borne, Aherne, Scannell, Kane, and Barron are in military hospitals at home.

